# A Diagnostic Quandary of Calvarial Tuberculosis: A Case Report

**DOI:** 10.31729/jnma.4319

**Published:** 2019-06-30

**Authors:** Sushan Homagain, Suraj Shrestha, Sushil Sah, Gopal Sedain

**Affiliations:** 1 Maharajgunj Medical Campus, Institute of Medicine, Maharajgunj, Kathmandu, Nepal; 2 Department of Neurosurgery, Tribhuvan University Teaching Hospital, Maharajgunj, Kathmandu, Nepal

**Keywords:** *calvaria*, *Mycobacterium*, *osteomyelitis*, *tuberculosis*

## Abstract

Primary Calvarial Tuberculosis, a rare entity of skull is even rarer after second decade of life in a healthy person without evidence of tuberculosis elsewhere in the body. Most of the cases are often misdiagnosed as osteomyelitis/syphilis/bony metastasis.

We report a case of primary skull tuberculosis in 26-year-old male with complains of headache and swelling in the right frontal region with no history of previous tuberculosis. The patient was operated and the histopathological examination of excised tissue was suggestive of tubercular pathology. The patient is doing well after anti-tubercular therapy.

Being a rare disease, tubercular osteomyelitis of skull bones is often missed and misdiagnosed due to lack of clinical suspicion and slow growth of mycobacterium cultures. Histopathological examination of biopsy material and demonstration of acid-fast bacilli in the pus are helpful for diagnosis and early management of the disease.

## INTRODUCTION

Tuberculosis (TB) is still endemic in developing countries with pulmonary TB being the most common. Calvarial Tuberculosis is a rare entity and has been reported in only 0.01% of all patients with mycobacterial infections.^1^ Calvarial TB in middle aged male with no evidence of tuberculosis elsewhere in body is very rare.^2^

It is interesting to note that, this case occurred in young, healthy man without any past history of tuberculosis. Here we report a case of primary calvarial tuberculosis. Very few cases have been reported from across the country.

## CASE REPORT

A 28-year-old male presented with a history of headache since 2 months and progressively increasing swelling over the right frontal region of skull for one month. The headache had been progressively increasing in severity for the last 20 days that was localized to right frontal region without radiation. The patient also had occasional fever without associated chills and rigor. There was no known history of trauma or weight loss. He did not have any form of TB in the past but was staying with a friend in a hostel who was taking anti-tuberculous therapy. The patient is a non-smoker and a social drinker.

**Figure 1.**
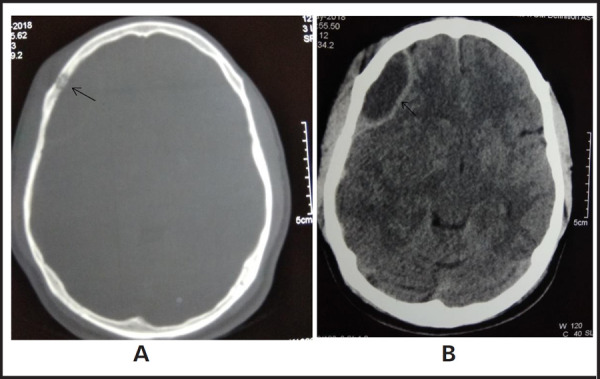
Plain Axial CT scan [(A) - Bone window (B)- Brain window] suggesting bone erosion and epidural and subgaleal collections.

On local examination, 5cm x 4cm soft, non-tender swelling was present on the right forehead. Local temperature was normal with no overlying skin changes. Swelling was non-pulsatile and non-expansible. On systemic examination, power was normal in all limbs, sensation and cranial nerves were intact. Signs of meningeal irritation were absent and plantar reflex was bilateral down going. Routine investigations along with non-contrast Computed Tomographic (CT) scan of the head was advised. The routine investigations were normal. However, CT scan demonstrated erosion of right frontal bone involving both inner and outer table with lentiform shaped extra-axial collection and soft tissue swelling in adjacent scalp - likely features of osteomyelitis. (Figure 1). Chest x-ray was normal.

The patient was admitted for surgical removal of epidural and subgaleal collections. The patient was started on Intravenous antibiotics (ceftriaxone 1gm BD, metronidazole 500mg TDS, and amikacin 750mg OD). Curvilinear incision was made at the right fronto- temporal region. Perioperative finding showed approximately 20ml of frank pus beneath the skin.

The underlying bone was soft with multiple pores and pus exudation from bone underneath. The pus was drained and sent for culture and sensitivity. The adjacent inflamed peri-cranium was sent for histopathological examination. Burr hole made at the infected site showed frank pus in the epidural space. IV antibiotics were continued. The patient recovered well post operatively.

**Figure 2.**
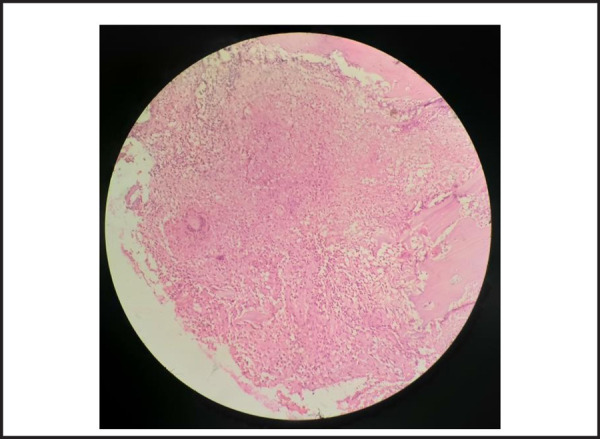
Histopathological examination showing giant cell, lymphocytes and caseous necrosis along with bone fragments at 40x magnification.

Histopathological examination showed well-formed granulomas with caseous necrosis and bony fragments. (Figure 2). Acid-fast bacilli were also seen which was consistent with tuberculosis. The patient was then started on Category I Anti tuberculous therapy [Isoniazid + Rifampin + Ethambutol + Pyrazinamide for two months (Intensive phase) and Isoniazid + Rifampin for 4 months (Continuation phase)] according to the national tuberculosis guideline.

**Figure 3.**
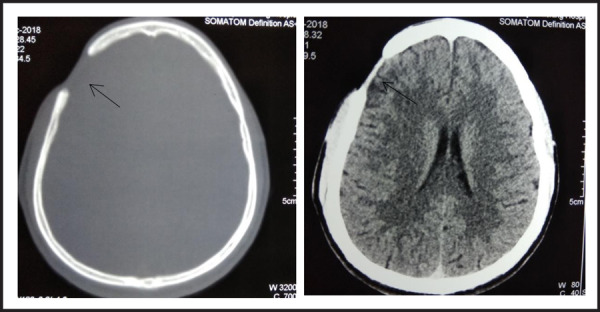
Plain Axial CT scan [(A) - Bone window (B) - Brain window] showing calvarial defect in right frontal bone without epidural and subgaleal collection.

On follow up, the symptoms had subsided. The patient was well at the time of writing, and had completed the course of therapy. CT head done on follow up after 1 month showed complete resolution of the abscess. (Figure 3).

## DISCUSSION

Although the incidence of tuberculosis has decreased in industrialized world, it still remains endemic in developing countries like Nepal. Skeletal Tuberculosis affects only about 1% of people with mycobacterial infection but only 0.1% to 3.7% of these are estimated to have skull bone tuberculosis making it a rare disease worldwide.^3,4^ But the incidence of skull bone tuberculosis is increasing due to malnutrition, poor socio-economic conditions and immunodeficiency states.^5^

Reid reported the first case of calvarial tuberculosis in 1842.^6^ Commonly the frontal and parietal bones, followed by occipital and sphenoid bones are involved due to high cancellous portion.^7^ Though there is no sex predilection, the disease primarily affects the younger population, with as many as 50% of patients below 10 years and 90% below 20 years but rarely among infants.^8^ Most cases of calvarial tuberculosis are secondary to pulmonary tuberculosis.^4,9,10^

But direct spread from face, orbit, paranasal sinuses and nasal mucosa have also been implicated.^11^ Primary calvarial tuberculosis without evidence of tuberculosis elsewhere in the body is still rarer.^12^

However, trauma to the skull leading to increased vascularity and decreased resistance can help unmasking latent mycobacterial infection.^13^ In our case no history of trauma was documented but unnoticed trauma which has escaped the patient's attention might have been the possible cause of primary tuberculosis. Moreover, there was also no evidence of any primary active focus of tuberculosis elsewhere in the body in our patient.

The presentation is highly varied. Most common presentations are fluctuant scalp swelling and a discharging sinus.^8,14^ Other presentations include low grade evening rise of fever, headaches, frank meningitis with seizure and other neurological deficits which are rare.^15^ In our case, swelling, occasional headache and fever were the only symptoms.

CT findings in calvarial tuberculosis is not very specific.^16^ Findings varies from extradural enhancing collection, calvarial destruction, sub galeal collection, sinus formation and sometime parenchymal involvement in various combination. CT scan of the brain helps in assessing the extent of bone destruction, scalp swelling, and degree of intracranial involvement. CT scans reveal extradural compression, if present, and help to rule out associated intradural lesions as tuberculomas or subdural empyemas.^1,7^ Chest radiographs are generally of little help-it was normal in our case. Contrast-Enhanced CT (CECT) is useful in diagnosis of Calvarial tuberculosis. It demonstrates the soft tissue swelling, the bony destruction and the extra-dural collection if any.^1^

Magnetic Resonance (MR) imaging, in most cases, leads to a conclusive diagnosis. Proton density and T2-weighted images show a high signal-intensity soft-tissue mass within the defect in bone. This may project into the subgaleal and/or epidural spaces and show peripheral capsular enhancement on the contrast-enhanced image.^17^

It is usually not possible to reach a conclusive diagnosis on the basis of imaging alone.^1^ Microbiologic or histologic confirmation is essential. Demonstration of Acid-fast bacilli in the pus smear by Ziehl-Neelsen stain and presence of caseous granuloma on histopathologic examination provides conclusive evidence of tuberculosis infection. A positive Mantoux test and raised ESR are important diagnostic clues for tuberculosis, but can be negative in 10% of patients.^18^ Positive serological tests like ELISA or PCR, if available, also contribute to the diagnosis.^1^

A clinical response to anti-tubercular treatment is an important criterion, especially in developing countries with high incidence of tuberculosis infection and limited access to advanced diagnostic techniques.^4,19^

Although there are reports that favor anti-tubercular therapy alone for the management but the combination of surgical debridement and treatment with antituberculosis drugs provide the best result.^13,18^ Early surgical intervention, microbiological and histopathological analysis of the resected specimen and medications are necessary for good outcomes.^1^ In developing country like Nepal where tuberculosis is rampant, a high degree of suspicion and awareness of this condition is necessary due to its varied presentation. Because of its rarity, the condition is often misdiagnosed as bacterial osteomyelitis / syphilis / histiocytosis / malignancy / aneurysmal bone cyst.^11,16,20^

Calvarial tuberculosis is an uncommon presentation of a common disease even in an endemic country. Cases can often be misdiagnosed as infectious osteomyelitis if culture/histopathological examination is not performed, often due to secondary infection. This leads to inappropriate treatment with antibiotics, which can escalate patient's expenses. Therefore, a high degree of suspicion along with imaging studies and histopathology/culture is required for a prompt diagnosis and management.
